# Oligomeric Aβ-induced synaptic dysfunction in Alzheimer’s disease

**DOI:** 10.1186/1750-1326-9-48

**Published:** 2014-11-14

**Authors:** Shichun Tu, Shu-ichi Okamoto, Stuart A Lipton, Huaxi Xu

**Affiliations:** Neuroscience and Aging Research Center, Sanford-Burnham Medical Research Institute, 10901 North Torrey Pines Road, La Jolla, CA 92037 USA

**Keywords:** Alzheimer’s disease, Synaptic loss, Aβ oligomers, Cognitive decline, Calcium, NMDA receptors, PSD-95, Mitochondrial dysfunction, Tau hyperphosphorylation, Aberrant neuronal network activity

## Abstract

Alzheimer’s disease (AD) is a devastating disease characterized by synaptic and neuronal loss in the elderly. Compelling evidence suggests that soluble amyloid-β peptide (Aβ) oligomers induce synaptic loss in AD. Aβ-induced synaptic dysfunction is dependent on overstimulation of *N*-methyl-D-aspartate receptors (NMDARs) resulting in aberrant activation of redox-mediated events as well as elevation of cytoplasmic Ca^2+^, which in turn triggers downstream pathways involving phospho-tau (p-tau), caspases, Cdk5/dynamin-related protein 1 (Drp1), calcineurin/PP2B, PP2A, Gsk-3β, Fyn, cofilin, and CaMKII and causes endocytosis of AMPA receptors (AMPARs) as well as NMDARs. Dysfunction in these pathways leads to mitochondrial dysfunction, bioenergetic compromise and consequent synaptic dysfunction and loss, impaired long-term potentiation (LTP), and cognitive decline. Evidence also suggests that Aβ may, at least in part, mediate these events by causing an aberrant rise in extrasynaptic glutamate levels by inhibiting glutamate uptake or triggering glutamate release from glial cells. Consequent extrasynaptic NMDAR (eNMDAR) overstimulation then results in synaptic dysfunction via the aforementioned pathways. Consistent with this model of Aβ-induced synaptic loss, Aβ synaptic toxicity can be partially ameliorated by the NMDAR antagonists (such as memantine and NitroMemantine). PSD-95, an important scaffolding protein that regulates synaptic distribution and activity of both NMDA and AMPA receptors, is also functionally disrupted by Aβ. PSD-95 dysregulation is likely an important intermediate step in the pathological cascade of events caused by Aβ. In summary, Aβ-induced synaptic dysfunction is a complicated process involving multiple pathways, components and biological events, and their underlying mechanisms, albeit as yet incompletely understood, may offer hope for new therapeutic avenues.

## Introduction

Alzheimer’s disease (AD) is the most common form of dementia among the elderly. It is clinically characterized by progressive memory loss and cognitive dysfunction, with the eventual inability to perform activities of daily living (ADLs). According to the Alzheimer’s Association ( http://www.alz.org), 5.4 million Americans are currently living with Alzheimer’s disease, and one in eight older Americans will develop AD. While drugs are available to temporarily improve memory and cognitive function or delay the progress of dementia, AD remains a devastating neurodegenerative disorder without any effective cure or disease-modifying therapy [1, 2]. Following diagnosis, AD patients generally survive from several years to 20 years, depending on age and other health conditions. Although AD is pathologically characterized by the presence of extracellular deposition of plaques comprised of Aβ peptide and neurofibrillary tangles (NFTs) comprised of hyperphosphorylated-tau protein, accumulating evidence suggests that these abnormal protein deposits are unlikely the causative events in AD as Aβ plaque or NFT volume poorly correlate with the severity of dementia. Instead, the degree of dementia in premortem patients correlates more closely with the level of soluble oligomers of Aβ species in postmortem brains, especially in hippocampal and cortex regions associated with learning and memory function [3–5]. Aβ peptides are proteolytic products of the amyloid precursor protein (APP) and are sequentially cleaved by β- and γ-secretases [6]. Although Aβ peptides of varying length are produced, Aβ_1-42_ is considered to be comparatively more amyloidogenic and readily assembles into soluble oligomers and consequent fibril deposits. Aβ oligomers, also termed as Aβ-derived diffusible ligands (ADDLs), are thought to induce synaptic loss and progressive cognitive decline in AD, whereas monomers and fibrillary aggregates may be more inconsequential to pathogenesis [7]. In addition, synaptic protein depletion [8–10] and synaptic loss [11, 12] are also found in the same regions in human AD or mild cognitive impairment (MCI) postmortem brains, and the level of synaptic reduction closely parallels the degree of premortem cognitive impairment. These studies suggest that oligomeric Aβ elevation and synaptic loss, rather than Aβ plaque load, may represent the best indicators of the severity of dementia or cognitive impairment in AD. Moreover, these findings imply that rescue of synapses could prove to be disease modifying in AD.

Using transgenic animal models of AD, additional studies suggest that synaptic loss is induced by pathological Aβ elevation [13]. Although the molecular mechanism is still not fully understood, it is generally believed that Aβ oligomers at pathological concentrations trigger (most likely via an indirect pathway) overstimulation of extrasynaptic NMDA receptors (eNMDARs), leading to aberrant redox events and Ca^2+^ upregulation. Subsequent activation of downstream signal transduction pathways trigger a cascade of pathological events leading to synaptic disruption and neuronal loss. These include increased oxidative/nitrosative stress and mitochondrial dysfunction with consequent bioenergetic compromise, leading to dysregulation of synaptic neurotransmission and abnormal neuronal network activity [7, 14]. A number of synaptic proteins have been proposed as potential Aβ-binding partners under pathological conditions and their interactions are believed to mediate Aβ-induced synaptic dysfunction. These proteins include, but are not limited to, α7-nicotinic acetylcholine receptors (α7nAChRs) [15], NMDARs [16, 17], mGluR5 [18], neurotrophin receptor p75^NTR^ [19], cellular prion protein (PrP^C^) [20], PSD-95 [21], glutamate transporter [22], ephrin type-B receptor 2 (EphB2) [23], and ephrin type-A receptor 4 (EphA4) [24, 25]. The involvement of some of these proteins in Aβ-mediated neurotoxicity will be discussed in the following sections.

### Oligomeric Aβ induces synaptic dysfunction in AD mice

AD or amnestic MCI patients usually have trouble with spatial orientation in their daily routine and perform poorly in clinically-designed, hippocampus-dependent memory and navigation tests [26–28]. Studies using animal models of AD have suggested that soluble oligomeric Aβ species are critical in initiating a pathogenic cascade leading to synaptic dysfunction, neuronal loss, and AD-like cognitive impairment [7]. The association between amyloidogenic Aβ species and age-dependent memory loss was first described in Tg2576 mice expressing a human APP695 transgene containing the Swedish mutation (K670N/M671L) [29]. These mice show elevated brain Aβ_1-42/43_ levels by ELISA and perform poorly in Morris water maze tests of spatial memory [29, 30]. The association between Aβ and cognitive impairment has also been documented in other transgenic (Tg) animal models of AD including 3XTg-AD mice, which express three human mutant AD gene variants (PS1M146V, APPSwe, and tauP301L) and develop progressive plaques and tangles [31, 32] and the human amyloid precursor protein-overexpressing (hAPP) J20 mouse model [33]. Synaptic dysfunction was also observed in these AD transgenic mice with aberrantly elevated levels of oligomeric Aβ and deficits in learning and memory (see selected reviews: [34–36]). Moreover, these studies showed that synaptic and cognitive impairments are associated with the elevation of soluble oligomeric Aβ species and usually evident prior to the appearance of plaques and tangles in the relevant brain regions.

NMDAR-dependent long-term potentiation (LTP) in hippocampus has attracted broad attention as an electrophysiological measurement of synaptic strength and plasticity in various AD models. LTP induction requires activation of NMDARs, which triggers a signaling cascade that induces the recruitment of AMPARs into the postsynaptic membrane [37]. On the other hand, NMDAR-dependent long-term depression (LTD) is an activity-dependent reduction in the efficacy of neuronal synapses, which is mediated, at least in part, by AMPAR endocytosis [37]. Multiple studies have shown that LTP is impaired in AD or Aβ-exposed wild-type hippocampus, and this impairment is dependent on NMDARs and downstream pathways [38–44]. In contrast to LTP, Aβ application enhances LTD [22, 45, 46], consistent with the notion that Aβ causes synaptic depression.

**Figure 1:**
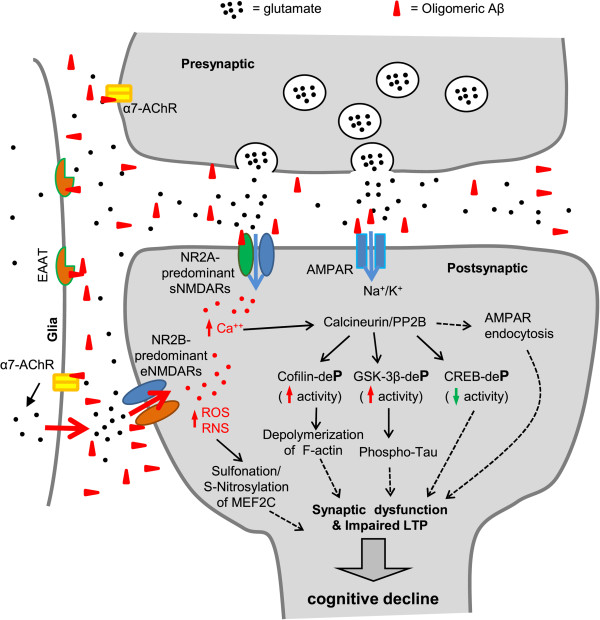
**Schematic diagram outlining mechanisms of oligomeric Aβ-induced synaptic dysfunction.** At pathological concentrations, Aβ oligomers may interact with multiple astrocytic, microglial, and neuronal synaptic proteins, including α7-AChRs and NMDARs, triggering a series of toxic synaptic events. These events include aberrant activation of NMDARs (especially NR2B-containing extrasynaptic NMDARs), elevated neuronal calcium influx, calcium-dependent activation of calcineurin/PP2B and its downstream signal transduction pathways, involving cofilin, GSK-3β, CREB, and MEF2. This results in aberrant redox reactions and severing/depolymerizing F-actin, tau-hyperphosphorylation, endocytosis of AMPARs, and eventually leads to synaptic dysfunction and cognitive impairment.

Emerging evidence suggests that Aβ-induced synaptic dysfunction is dependent on NMDAR-mediated activity and occurs via aberrant redox events as well as elevation of cytoplasmic Ca^2+^ and activation of downstream pathways involving Ca^2+^-dependent protein phosphatase calcineurin/PP2B and protein phosphatase 2A (PP2A) (Figure 1) [47–49]. Concerning the Ca^2+^-dependent events, upon activation, calcineurin further activates or inactivates its target proteins via dephosphorylation. For example, dephosphorylation and activation of the actin filament severing protein cofilin by calcineurin result in dendritic spine loss, which can be rescued by overexpression of the inactive cofilin phosphomimetic S3D [48]. Interestingly, a recent study reported that Aβ oligomers interact with murine PirB (paired immunoglobulin-like receptor B) and its human ortholog LilrB2 (leukocyte immunoglobulin-like receptor B2) with nanomolar affinity. This interaction enhanced cofilin signaling and contributed to memory loss in AD transgenic mice [50]. Aβ-induced synaptic degeneration also involves surface removal and endocytosis of AMPARs [45]. In support of this, surface AMPARs are downregulated through endocytosis in wild-type neurons rapidly upon Aβ-application [51] and in AD transgenic mice [52]. Concurrently, AMPAR-mediated synaptic currents are also downregulated in AD double knock-in (mutant APP and PS1) transgenic mice [53]. Aβ-induced AMPAR endocytosis or surface removal is dependent on the activation of calcineurin/PP2B [54] and requires downregulation of Ca^2+^/calmodulin-dependent protein kinase II (CaMKII) [55]. Similar to AMPARs, Aβ can induce surface removal or endocytosis of NMDARs, which is mediated by dephosphorylation of NR2B (GluN2B), an NMDAR subunit, at p-Tyr1472 by the tyrosine phosphatase STEP [56, 57]. Interestingly, the apoptotic effector component caspase-3 has also been suggested to play a role in synaptic plasticity and its activation is required in AMPAR removal and consequent LTD induction [58]. Activation of caspase-3 has been further shown to trigger early synaptic dysfunction in AD transgenic mice [59]. This is consistent with the observation that caspase-3 is enriched in postsynaptic densities [60]. In addition to ionic glutamate receptor NMDARs and AMPARs, metabotropic glutamate receptors (mGluRs) at extrasynaptic or perisynaptic sites have also been shown to play an important role in Aβ-induced synaptic dysfunction [46], likely activated by pathologically elevated glutamate at extrasynaptic sites [22].

It has also been reported that pathological concentrations of Aβ oligomers disrupt glutamate uptake, thus increasing glutamate levels to enhance LTD [22] or impair LTP [61]. Consistent with these results, the levels of both glutamate transporters EAAT1 and EAAT2, which are responsible for the majority of glutamate uptake in glial cells, are downregulated in the hippocampus of AD patients [62]. Due to impaired glutamate uptake, glutamate spills out of synapses and accumulates in the extrasynaptic spaces, thereby inducing overactivation of NR2B-predominant eNMDARs [61]. In addition to attenuated glutamate uptake, oligomeric Aβ increases glutamate levels by triggering aberrant astrocyte glutamate release and accumulation within the extrasynaptic space thereby overactivating eNMDARs, which results in synaptic damage via a variety of aberrant transcriptional cascades and redox-mediated posttranslational modifications [63–69]. These eNMDAR-mediated pathways include hyperactivation of neuronal nitric oxide synthase (nNOS) and Ca^2+^-overload of mitochondria, generating excessive reactive nitrogen and oxygen species (RNS/ROS). This excess results in aberrant oxidation reactions, such as S-nitrosylation and sulfonation, on various proteins, thereby disrupting their normal activity. One example is illustrated by excessive S-nitrosylation or sulfonation of the transcription factor MEF2C, thereby adversely affecting downstream effectors of neuronal survival and adult neurogenesis [68, 70]. Importantly, memantine, an FDA-approved, uncompetitive NMDAR open-channel blocker, at proper concentration, preferentially blocks pathologically-stimulated eNMDARs over physiologically-activated synaptic NMDARs (sNMDARs) [71]. Indeed, application of memantine, and to a greater degree its improved derivative NitroMemantine [72], has been shown to mitigate Aβ-induced synaptic dysfunction and cognitive deficits [63, 73]. Taken together, these studies depict an alternative mechanism underlying Aβ-induced synaptic dysfunction based on glutamate-mediated hyperexcitability and synaptic excitotoxicity: Aβ-induced accumulation of excessive glutamate in the extrasynaptic space can occur through disruption of the glutamate uptake system or by triggering astrocytic glutamate release, which in turn aberrantly activates eNMDARs and induces synaptic dysfunction (Figure 1).

NMDAR and AMPAR function at postsynaptic sites are influenced by interaction with PSD-95, an important postsynaptic scaffolding protein that plays a critical role in protein assembly, synaptic development and neural plasticity [74, 75]. Under pathological conditions however, synaptic PSD-95 levels are decreased in AD postmortem brains, and the degree of reduction correlates with both the level of Aβ oligomers and the severity of dementia [21, 76–78]. Similarly, PSD-95 is also reduced in AD transgenic mouse neurons or neurons exposed to Aβ oligomers, concomitant with dendritic spine loss and surface AMPAR removal [51, 52, 79]. These results suggest that synaptic disruption of PSD-95 may play a role in the pathogenesis of AD. Consistent with this idea, it has been shown by co-immunoprecipitation that Aβ interacts with PSD-95 and is co-localized with PSD-95 specifically at excitatory synapses in human postmortem AD brain as well as in cultured murine neurons exposed to Aβ oligomers [21, 80]. Therefore, it is possible that Aβ may interact with PSD-95 directly to cause synaptic damage under pathological conditions. Interestingly, overexpression of α1-takusan, a PSD-95-binding and synaptic-stabilizing protein [81], decreases Aβ-induced synaptic damage, protecting from dendritic spine loss, decreased synaptic expression of PSD-95, and downregulated AMPAR-mediated synaptic currents in cultured neurons [82]. Further evidence has shown that increased PSD-95 expression in hippocampus after treatment with the Hsp90 inhibitor 17-AAG can improve cognitive function in an animal model of AD, apparently via synaptic enhancement [83]. Therefore, molecules that modulate the integrity of PSD-95 and improve synaptic functions may have therapeutic potential for reducing Aβ-induced synaptic injury and cognitive impairment in AD.

A number of other synaptic proteins have been proposed to be putative receptors for Aβ oligomers, and play important roles in oligomeric Aβ-induced synaptic dysfunction and cognitive impairment. For example, soluble Aβ oligomers can bind with nanomolar affinity to PrP^C^, and PrP knockout or anti-PrP antibodies can rescue oligomeric Aβ-induced synaptic dysfunction and spatial memory. These results suggest that prion proteins play an important role in AD pathogenesis [20]. The α7nAChR modulates calcium homeostasis and release of the neurotransmitter glutamate, two important parameters involved in learning and memory. Endogenous Aβ species have been reported to bind to α7nAChRs with nanomolar affinity in co-immunoprecipitation experiments on human AD postmortem brain extracts [15]. This interaction consequently triggers α7nAChR-dependent NMDAR endocytosis leading to synaptic and cognitive dysfunction [15, 56]. The ephrin family of receptor tyrosine kinases has also been found to be a potential receptor that interacts with Aβ oligomers under pathological conditions. It has been shown that Aβ oligomer binding to EphB2 induces its degradation, leading to impairments in NMDAR-mediated synaptic activity and cognitive function [23]. Conversely, EphB2 overexpression reverses deficits in NMDAR-dependent LTP and cognitive impairments in AD Tg mice [23]. Recently, ephrin A4 (EphA4), another ephrin receptor family member, was also identified as a putative Aβ receptor [24, 25]. In contrast to EphB2 degradation, interaction with Aβ activates EphA4, leading to suppression of LTP and spine loss in AD transgenic mice. Application of either EphA4 shRNA or EphA4 inhibitors/antagonists has been reported to rescue these deficits, suggesting that EphA4 activation plays a critical role in Aβ-induced synaptic dysfunction [24, 25].

### Alternative pathways contribute to Aβ-induced synaptic and neuronal loss in AD

Aβ-induced synaptic dysfunction can also be mediated by molecules and their downstream pathways that are not directly associated with NMDA receptor-mediated activities. It has been shown that Aβ can bind to the low-affinity p75 neurotrophin receptor (p75^NTR^) and activate its death domain to induce apoptosis [19]. Moreover, surface expression level of p75 is upregulated in SH-SY5Y neuroblastoma cells after exposure to Aβ oligomers and in hippocampal neurons in AD transgenic mice [84]. Consistent with these animal studies, the level of membrane-associated p75 in hippocampus was significantly higher in human postmortem AD brains compared to age-matched controls [85]. Interestingly, APP cleavage is differentially regulated by the neurotrophin high-affinity receptor TrkA and the low-affinity receptor p75^NTR^; p75^NTR^ promotes whereas TrkA decreases APP β-cleavage [86]. Therefore, aberrantly upregulated p75^NTR^ together with TrkA downregulation in aged brains results in increased Aβ generation [87]. In addition, p75^NTR^ may also enhance Aβ production via its ability to stabilize BACE1 or β-secretase through the activation of sphingomyelinase and consequent ceramide production [87, 88].

In addition to neurotrophin receptors, insulin and insulin-like growth factor receptors and their cognate signaling pathways play a critical role in synaptic plasticity and cognitive function by affecting both excitatory and inhibitory synaptic activity [89, 90]. Growing evidence suggests that AD may represent a metabolic disease of the brain associated with brain insulin and insulin-like growth factor-I (IGF-I) resistance and deficiency. Impaired insulin signaling may contribute to dysregulation of downstream pro-survival pathways, including decreased signaling mediated by PI3K, Akt, and Wnt/β-catenin; moreover, disrupted insulin-related signaling may enhance pathogenic pathways such as GSK-3β to trigger tau hyperphosphorylation [91, 92]. Therefore, disrupted components of brain insulin signaling pathways may represent potential therapeutic targets in AD [91, 92]. It has been suggested that both extracellular and intracellular Aβ oligomers contribute to neuronal dysfunction. Intracellular Aβ inhibits insulin receptor signaling by interfering with the interaction between phosphoinositide-dependent kinase (PDK) and Akt, thus inhibiting Akt activation and abolishing insulin-mediated neuroprotection [93]. Extracellular Aβ oligomers or ADDLs bound to synaptic sites can induce removal of surface insulin receptors and contribute to synaptic loss, which can be rescued by insulin treatment [94]. Encouraging results obtained from intranasal insulin therapy in aged adults [95] as well as in AD and MCI patients [96] support the idea that insulin signaling is disrupted during normal aging and in clinical cases of dementia.

Furthermore, Wnt family members promote synaptic formation and regulate synaptic function by binding to receptors of the Frizzled (Fz) and low-density lipoprotein-related protein (LRP) families on the cell surface to activate either β-catenin-dependent canonical signaling (Wnt/β-catenin) or β-catenin-independent non-canonical signaling pathways; the latter pathways include the Wnt/PCP and Wnt/Ca^2+^ cascades [97]. Multiple Wnt signaling components are dysregulated in AD and such impairments are likely to contribute to synaptic dysfunction and cognitive decline in AD. Wnt co-receptor LRP6 variants suppress Wnt signaling activity and are associated with late-onset AD [98]. An endogenous Wnt inhibitor, Dickkopf-1 (Dkk1), which disrupts Wnt-induced Fz/LRP complex formation [99], is increased in human postmortem AD brains [100] and mouse model AD brains [101], where Dkk1 has been found to co-localize with active GSK-3 and phospho-tau. Additional studies suggest that Dkk1 levels increase after Aβ oligomer exposure [102]. Dkk1 upregulation appears to be required for Aβ-induced synaptic loss since synaptic damage as well as tau phosphorylation are abolished by either Dkk1 knockdown [100] or Dkk1 blocking antibodies [102]. Interestingly, it has been shown that Aβ may directly interact with Fz receptors of Wnt ligands [103] although the significance of this interaction remains to be determined. Taken together, these studies suggest that dysregulated Wnt signaling contributes to Aβ-induced synaptic loss in AD, which raises the possibility that Wnt signaling components may represent potential therapeutic targets in AD.

### Aberrant neuronal network activity and seizures in AD

Epileptic seizures were once considered to be rare or an epiphenomenon in AD. However, accumulating evidence suggests that increased seizure activity may be the consequence of a disrupted neuronal network, contributing to cognitive decline and the onset of dementia [104]. It has been estimated that 10 to 22% of AD patients experience at least one episode of an unprovoked seizure [105]. However, it is likely that these numbers are underestimates for the following reasons: (1) AD patients with dementia may not recall having seizures if they were unwitnessed, and (2) some types of seizure activity, including complex partial seizures, manifest symptoms such as confusion or delirium, which are similar to those normally seen in AD patients, and therefore may go undetected. Prior observational studies have shown that the occurrence of a first unprovoked seizure in patients 55 years or older is significantly greater in AD and other dementias compared to the general population [106]. The increased incidence can be as high as 87-fold in AD patients with early-onset FAD [107]. Consistent with this result, a more recent study found that FAD mutations in *APP*, *PSEN1*, and *PSEN2* are all associated with higher risk for a first unprovoked seizure [108]. These results suggest a tight association between epileptic seizures and genetic mutations that cause aberrant expression of Aβ oligomers and induce early-onset AD.

Mechanistic insight into this abnormal excitatory phenomenon was obtained in studies using AD transgenic mice expressing human mutant APP and presenilin genes. Several early studies showed that AD transgenic mice expressing human Aβ fragments [109] or APP mutations [110, 111] exhibit increased spontaneous seizure activity, although these unprovoked seizure events are rare and often ignored. Detection of subtle seizure phenotypes (or nonconvulsive seizures) and aberrant neuronal network activities have been made possible through the use of video-electroencephalography (EEG) monitoring in AD transgenic mice expressing FAD mutations [112–114]. Acute application of levetiracetam (LEV or Keppra®), an antiepileptic drug, can effectively suppress abnormal EEG spike activity, and chronic treatment with LEV can even reverse AD-like phenotypes including synaptic dysfunction, hippocampal remodeling, and learning and memory deficits in human APP transgenic mice [115]. Therefore, antiepileptic drugs, such as LEV, that suppress the aberrant electrical activity that contributes to seizures may provide an alternative approach to the treatment of AD.

### Aβ-induced tau hyperphosphorylation and its role in synaptic loss

The presence of hyperphosphorylated tau-enriched neurofibrillary tangles is one of the classical pathological hallmarks of AD. Tau is a microtubule-associated protein (MAP) that was originally identified as an important protein for microtubule (MT) assembly [116] and for stabilization of the MT network [117]. Under pathological conditions, tau becomes hyperphosphorylated and disassociated from microtubules, subsequently forming soluble aggregates, insoluble filaments, and eventually neurofibrillary tangles (NFTs) in affected brain regions. This pathology occurs not only in AD but also in several other neurological disorders, which are collectively termed tauopathies [118, 119]. Phosphorylated tau (p-tau) colocalizes with Aβ in synaptic terminals from both postmortem AD brain [120, 121] and transgenic mouse AD brain [122]. These prior studies have shown that expression of p-tau in synaptic terminals correlates with Aβ levels, and increased p-tau expression also correlates with a reduction in total synapse number. A causal association between oligomeric Aβ exposure and p-tau formation has been demonstrated in several studies. For example, Talantova et al. [63] reported that oligomeric Aβ caused astrocytic glutamate release, which in turn activated extrasynaptic NMDARs, resulting in increased p-tau levels. Additional studies have shown that Aβ-induced synaptic loss is tau-dependent since tau deletion or reduction can rescue Aβ-induced synaptic loss and cognitive impairment in AD transgenic mice [123–126]. Moreover, a recent study characterized interactions between oligomeric Aβ and p-tau in both human and animal AD brains by co-immunoprecipitation and immunohistology, and this interaction progressively increased with disease progression [127]. It is thus possible that pathological interactions between oligomeric Aβ and p-tau are important intermediate steps in Aβ-induced synaptic loss and neuronal damage. Although previously known as an axonal protein, tau is also expressed in dendrites and in the postsynaptic density, albeit at much lower levels [124]. Depletion of dendritic or postsynaptic tau prevents abnormal postsynaptic targeting of the tyrosine kinase Fyn and rescues impaired learning and memory function in AD transgenic mice, suggesting a vital role of dendritic and postsynaptic tau in Aβ-induced synaptic loss [124]. In cultured neurons, Aβ-induced tau hyperphosphorylation and dendritic disruption can be attenuated by anti-Aβ antibodies or tau reduction using RNAi-targeting strategies [128–130]. Therefore, it is possible that Aβ-induced tau hyperphosphorylation is an important intermediate event that leads to synaptic dysfunction. Indeed, only pseudohyperphosphorylated tau, which mimics hyperphosphorylated tau, but not phosphorylation-deficient tau, which mimics regular tau, is mislocalized and accumulated in dendritic spines [131]. Consequently, hyperphosphorylated tau that is mislocalized to dendritic spines has been reported to induce synaptic dysfunction by impairing AMPAR surface expression and synaptic transmission [131]. Consistent with this finding, tau deletion or inhibition of tau hyperphosphorylation using a glycogen synthase kinase 3β (GSK-3β) inhibitor can prevent Aβ-induced impairment of LTP [132]. GSK-3β-mediated tau phosphorylation and Aβ production can also be reduced by RPS23R1 protein via activation of the adenylate cyclase/cAMP/PKA pathway. In turn, this pathway leads to synaptic enhancement and improved AD pathology [133]. Tau-dependent synaptic dysfunction may also involve the tyrosine kinase Fyn [124]. Pseudohyperphosphorylated tau binds Fyn more tightly than wild-type tau, thus increasing Fyn activity and leading to synaptic damage [134]. Intriguingly, a study using human AD induced pluripotent stem cell (iPSC)-derived neurons has suggested that increased tau phosphorylation at Thr231 is mediated by β-secretase activity [135]. This finding raises the possibility that Aβ-induced tau pathology and synaptic damage can be mediated by an alternative, non-Aβ-mediated pathway. Thus, future in-depth studies will determine the role of dendritic/postsynaptic tau and its hyperphosphorylation in Aβ-induced synaptic loss.

### Aβ-induced mitochondrial dysfunction and synaptic loss in AD

Under physiological conditions, mitochondria provide much of the energy that is required to maintain normal synaptic activity and plasticity [136]. However, under pathological conditions, mitochondrial impairment has been suggested to be an important early event contributing to synaptic loss and neurodegeneration in AD [137–139]. In human postmortem brains, Aβ has been reported to accumulate aberrantly in mitochondria with abnormal morphology, suggesting that Aβ may influence mitochondrial morphogenesis [140–143]. Wild-type neurons exposed in vitro to oligomeric Aβ or AD transgenic neurons in vivo exhibit excessive mitochondrial fission (mitochondrial fragmentation), a sign of impairment in mitochondrial dynamics, further implicating Aβ in mitochondrial dysregulation [144–147]. Therefore, it is possible that Aβ at pathological concentrations can trigger mitochondrial impairment, which in turn leads to bioenergetics compromise, synaptic starvation and damage in AD. Consistent with this hypothesis, mitochondrial impairment caused by oligomeric Aβ or toxic factors generated downstream of Aβ, such as RNS/NO, occurs prior to synaptic and neurite injury [148] and precedes AD pathology [149]. It has been suggested that abnormal interactions between Aβ and the mitochondrial fission protein dynamin-related protein 1 (Drp1) play an important role in mitochondrial dysfunction and synaptic damage in AD [150]. Additional evidence suggests that Aβ-induced mitochondrial impairment is likely mediated by abnormal interaction between oligomeric Aβ and mitochondrial matrix protein ABAD [151] or cyclophilin D (CypD) [152]. It has also been suggested that appoptosin, a glycine/5-amino-levulinic acid transporter mediating heme synthesis in mitochondria, is involved in Aβ-induced neurodegeneration by inducing ROS release and resultant apoptosis under pathological conditions; these pathogenic processes can be prevented by appoptosin downregulation [133].

Synaptic mitochondria, or mitochondria residing in synapses, are usually older, and thus much more vulnerable to persistent insults such as Aβ compared to nonsynaptic mitochondria. Aβ species reportedly also accumulate at much higher levels in synaptic mitochondria compared to nonsynaptic mitochondria [153, 154]. Accordingly, synaptic mitochondria show greater susceptibility to damage compared to nonsynaptic mitochondria in AD transgenic mice [153, 154]. Mitochondrial function such as respiratory rate, ROS production, membrane potential, and cytochrome c oxidase activity are compromised more severely in synaptic mitochondria than in nonsynaptic mitochondria [153, 154]. The degree of mitochondrial impairment is also region-specific. Greater damage is found in areas related to learning and memory such as the hippocampus and cortex, while only moderate damage is found in other brain regions [154].

Tau pathology strongly correlates with mitochondrial impairment [125, 130, 155–158], suggesting that tau may play a role in Aβ-induced mitochondrial dysfunction. It has been shown using immunoprecipitation and immunofluorescence that hyperphosphorylated tau abnormally interacts and colocalizes with the mitochondrial fission protein Drp1 in postmortem AD brains [158]. In the same brain specimens, elevated levels of Drp1 and mitochondrial fragmentation have also been identified [150]. These studies suggest that hyperphosphorylated tau is associated with Aβ-induced mitochondrial dysfunction, likely through its abnormal interaction with Drp1 in AD neurons. Consistent with this notion, Aβ application in cultured neurons results in elevated expression of mitochondrial fission proteins Drp1 and Fis1 and reduced expression of fusion proteins Mfn1, Mfn2, and Opa1, consistent with mitochondrial fragmentation phenotypes observed in AD [146]. Additional recent evidence also supports a role for hyperphosphorylated tau in mitochondrial impairment. For example, overexpression of the postsynaptic protein α1-takusan inhibits Aβ-induced tau hyperphosphorylation and prevents Aβ-induced mitochondrial fragmentation in cultured neurons [82].

**Figure 2:**
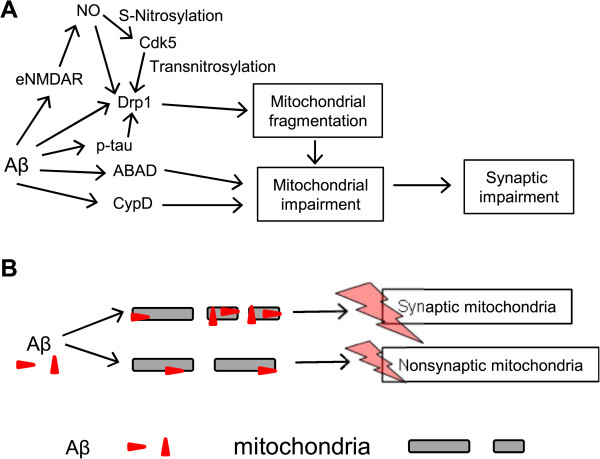
**Mitochondrial impairment and synaptic dysfunction in AD. (A)** Under pathological conditions, Aβ oligomers directly or indirectly affect mitochondrial fission proteins such as Drp1 and mitochondrial matrix proteins, including ABAD and CypD. Such interactions may mediate Aβ-induced mitochondrial fragmentation with consequent bioenergetics failure and resulting synaptic loss. **(B)** Synaptic mitochondria are more vulnerable to pathological toxins than non-synaptic mitochondria. Aβ is also more likely accumulated in synaptic mitochondria than in non-synaptic mitochondria, thus enhancing damage on synaptic mitochondria.

Moreover, pathological redox reactions of mitochondrial proteins triggered by oligomeric Aβ, in part mediated by aberrant eNMDAR stimulation as discussed above, have been described. These reactions include S-nitrosylation of Drp1 (to form SNO-Drp1), resulting in excessive mitochondrial fission with consequent bioenergetic compromise and hence synaptic damage [64]. Interestingly, Cdk5 has been shown to act as a transnitrosylase, transferring NO from Cdk5 to Drp1 (as opposed to the classical role of Cdk5 as a kinase). Transnitrosylation hyperactivates Drp1 under these disease conditions, and, importantly, S-nitrosylation of Cdk5 is triggered by oligomeric Aβ peptide [67]. Thus, the initial nitrosylation of Cdk5 may represent in inciting event in mitochondrial fragmentation, with resulting bioenergetic failure and synaptic loss. Figure 2 summarizes our current understanding of Aβ-induced mitochondrial impairment as discussed above.

## Conclusions

Accumulating evidence suggests that oligomeric Aβ plays a central role in synaptic dysfunction and cognitive decline in AD. Studies using animal models have revealed that Aβ-induced synaptic dysfunction involves multiple pathological events and various integral signaling systems, including glutamate receptors and their downstream pathways, abnormal elevation of extrasynaptic glutamate levels and subsequent eNMDAR-mediated excitotoxicity, tau hyperphosphorylation, and impaired mitochondria. However, the relevance of these studies to AD in human remains debatable. Furthermore, the detailed molecular mechanisms underlying these events are still not fully understood. Several fundamental questions remain, such as the role of tau phosphorylation in Aβ-induced synaptic dysfunction, the physiological relevance of Aβ-binding partners or receptors in neuronal degeneration, and even the direct role of Aβ itself in AD. Since a large variety proteins and distinctive pathways may be involved in the pathogenesis of AD, there may be no definitive treatment that can ubiquitously treat all AD patients. Indeed, the complexity of AD is exemplified by a diverse set of genetic mutations associated with AD. If this holds true, personalized drug candidates may need to be developed to cater to various genetic profiles, severity in cognitive decline, and other environmental factors. These challenges remain daunting. However, development of new technologies to treat dysregulated molecular pathways downstream of Aβ and phospho-tau may enable us to utilize our emerging knowledge of these pathways in order to develop novel strategies in the treatment and prevention of AD.

## Abbreviations

ADDLs: Aβ-derived diffusible ligands
ADLs: Activities of daily living
α7nAChRs: α7-nicotinic acetylcholine receptors
AD: Alzheimer’s disease
AMPARs: AMPA receptors
Aβ: Amyloid-β peptide
APP: Amyloid Precursor Protein
CaMKII: Ca^2+^/calmodulin-dependent protein kinase II
Cdk5: Cyclin-dependent kinase 5
CypD: Cyclophilin D
Dkk1: Dickkopf-1
Drp1: Dynamin-related protein 1
EEG: Electroencephalography
eNMDARs: Extrasynaptic NMDARs
EAAT1: Excitatory Amino Acid Transporter 1
EAAT2: Excitatory Amino Acid Transporter 2
GSK-3β: Glycogen synthase kinase 3β
hAPP: Human amyloid precursor protein-overexpressing
iPSC: Induced pluripotent stem cell
LTD: Long-term depression
LilrB2: Leukocyte immunoglobulin-like receptor B2
IGF-I: Insulin-like growth factor-I
LTP: Long-term potentiation
LRP: Low-density lipoprotein-related protein
mGluRs: Metabotropic glutamate receptors
MT: Microtubule
MCI: Mild cognitive impairment
NFTs: Neurofibrillary tangles
nNOS: Neuronal nitric oxide synthase
NMDARs: *N*-methyl-D-aspartate receptors
PirB: Paired immunoglobulin-like receptor B
p-tau: Phospho-tau
PDK: Phosphoinositide-dependent kinase
PrP^C^: Prion protein
PKA: Protein kinase A
PP2A: Protein phosphatase 2A
PP2B: Protein phosphatase 2B
RNS: Reactive nitrogen species
ROS: Reactive oxygen species
sNMDARs: Synaptic NMDARs
Tg: Transgenic.
